# Assessment of Iodine Status among Pregnant Women and Neonates Using Neonatal Thyrotropin (TSH) in Mainland China after the Introduction of New Revised Universal Salt Iodisation (USI) in 2012: A Re-Emergence of Iodine Deficiency?

**DOI:** 10.1155/2019/3618169

**Published:** 2019-10-07

**Authors:** Hang Zhou, Zheng Feei Ma, Yiming Lu, Binyu Pan, Jian Shao, Liya Wang, Yanyan Du, Qihua Zhao

**Affiliations:** ^1^Clinical Medical College, Yangzhou University, Yangzhou 225009, Jiangsu Province, China; ^2^Department of Clinical Nutrition, Northern Jiangsu People's Hospital, Yangzhou 225001, Jiangsu Province, China; ^3^Department of Health and Environmental Sciences, Xi'an Jiaotong-Liverpool University, Suzhou 215123, Jiangsu Province, China; ^4^Department of Orthopedics, Northern Jiangsu People's Hospital, Yangzhou 225001, Jiangsu Province, China; ^5^Department of Clinical Nutrition, The First People's Hospital of Wujiang District, Suzhou 215200, Jiangsu Province, China

## Abstract

Iodine deficiency during pregnancy can cause iodine deficiency disorders (IDD). However, it is unclear about iodine and thyroid status of Chinese pregnant women and neonates after the implementation of the revised universal salt iodisation (USI) level in 2012. Therefore, the aim of the cross-sectional study was to determine iodine nutrition and thyroid status among pregnant women and their neonates in China after the implementation of USI. Medical records of pregnant women and neonates in Northern Jiangsu People's Hospital between January 2016 and December 2017 were reviewed and included. We included 3060 mother-and-newborn pairs in the study. Mean age of participants was 28.2 ± 4.1 years. TSH, FT3, and FT4 of participants were within normal reference range. The overall mean neonatal TSH, birth weight, and prevalence of low birth weight (LBW) were 4.86 ± 2.06 mIU/L, 3358 ± 455 g, and 3.2%, respectively. The prevalence of neonatal TSH values >5 mIU/L was 29.3%, suggesting iodine deficiency in the region. In conclusion, our results indicated iodine deficiency in the region, according to the neonatal TSH cutoff recommended by WHO/UNICEF/IGD. More efforts are urgently required to improve iodine status of pregnant women in the region in order to prevent a re-emergence of iodine deficiency.

## 1. Introduction

Iodine is needed by the thyroid gland for the production of thyroid hormones [1]. Inadequate iodine intake can cause iodine deficiency which is one of the most common nutrient deficiencies worldwide affecting ∼30% of the world's population [1]. Iodine deficiency is associated with a spectrum of morbidities which is referred to as iodine deficiency disorders (IDD) [2]. In addition, iodine deficiency during pregnancy is associated with poor neurocognitive development in neonates [2]. Therefore, WHO/UNICEF/IGD [3] recommended that it is important to monitor iodine status in pregnant women using appropriate biomarkers of iodine status.

One of the biomarkers that can be used to assess iodine deficiency in a population is thyroid-stimulating hormone (TSH) [1], although TSH is commonly used in neonatal screening for congenital hypothyroidism [4, 5]. TSH is a 2-chain 28-kDa glycoprotein hormone [6]. Since TSH is determined primarily by the concentration of circulating thyroid hormones which reflects dietary iodine intake, TSH can be used to indicate iodine nutrition and thyroid function [4, 7]. In addition, TSH is regarded as a sensitive biomarker of iodine status in neonates [8]. This is because the low iodine content in the neonatal thyroid has a high iodine turnover, which requires increased TSH stimulation [3]. In addition, the high iodine turnover in neonatal thyroid is also exaggerated in iodine deficiency [3]. Therefore, the prevalence of neonates with elevated TSH can be used to indicate the degree of iodine deficiency in a given region [3]. In addition, it is also proportional to the severity of iodine deficiency during the period of pregnancy [3].

Jiangsu is a province located in eastern China with a population of about 79 million [9]. During the early 1980s, endemic goitre had been long recognized as a public health problem in Jiangsu. The prevalence of goiter among school-aged children was 25.5%, and mean urinary iodine concentration (UIC) was 76 *μ*g/L [10]. In addition, the mean iodine content in the drinking water was 7 *μ*g/L, suggesting low iodine content. Therefore, it was suggested that the iodine deficit was caused by environmental factors. In 1985, some counties in Jiangsu had initiated salt iodisation to combat IDD. In 1995, the implementation of mandatory universal salt iodisation (USI) at 50 mg iodine/kg salt was started in whole Jiangsu Province [10]. Since 1996, more than 90% of the population in Jiangsu have access to iodised salt [11]. Subsequently, Jiangsu has eliminated IDD in the population since 2001 [9]. In addition, the Chinese government had adjusted the iodine content in iodised salt three times to 25 mg iodine/kg salt (range: 18–33 mg iodine/kg salt) in order to ensure that the iodine nutrition of the population is maintained at an adequate level with reference to the results obtained from the IDD Surveillance Report of China [12].

The implementation of the new salt iodisation level might put some vulnerable groups such as pregnant women and neonates at risk of IDD [13]. This is because they require a higher iodine intake to ensure proper maternal thyroid function and fetal neurocognitive development [2]. Published data from China have indicated iodine sufficiency in the general population [14]. However, iodine deficiency has been reported in some provinces such as Xinjiang and Sichuan [14] and more recently, in Zhejiang [15–17]. Most of these studies only included pregnant women but not neonates [15–17]. In addition, most of these studies did not assess longer-term biomarkers of iodine status such as TSH [15–17], which can provide the information of thyroid status and iodine nutrition within weeks or months.

Therefore, the aim of the cross-sectional study was to determine iodine nutrition and thyroid status among pregnant women and their neonates in Jiangsu, China.

## 2. Methods

### 2.1. Study Population and Settings

The study was based on the data obtained from the pregnant women and their neonates in Northern Jiangsu People's Hospital, Yangzhou, Jiangsu, China from January 2016 to December 2017. To be eligible in the study, pregnant women must be aged ≥18 years with normal singleton pregnancy and no history of thyroid disease. Pregnant women who developed maternal and neonatal adverse outcomes were excluded from the survey. All data collection took place on the hospital premises.

Our retrospective review of the medical records of pregnant women and neonates was approved by the Ethics Committee of the Northern Jiangsu People's Hospital (reference no. 2018063). In addition, our study protocols were conducted according to the Declaration of Helsinki.

### 2.2. Sociodemographic Data Collection and Anthropometric Measurement

The information regarding the sociodemographics of pregnant women including age was recorded by the doctors during their hospital visits. The body weight and height of the pregnant women were measured to the nearest 0.1 cm and 0.01 kg, respectively. Their body mass index (BMI) was calculated by dividing body weight in kilograms (kg) by the height in meters (m) squared. BMI was categorised according to the recommended criteria for Chinese adults proposed by the Working Group on Obesity in China which were as follows: underweight, <18.5 kg/m^2^; normal weight, 18.5–23.9 kg/m^2^; overweight, 24.0–27.9 kg/m^2^; and obese, ≥28.0 kg/m^2^ [18–20].

### 2.3. Biochemical Measurements

Overnight fasting blood samples of pregnant women were obtained for the determination of thyroid-stimulating hormone (TSH), free triiodothyronine (FT3), and free thyroxine (FT4) during their hospital visits. Heel-prick blood samples of newborns were also collected using filter cards on day 3 after birth for the determination of neonatal TSH. In addition, the birth weight of newborns was also measured. In order to determine the general well-being of newborns at delivery, the appearance, pulse, grimace, activity, and respiration (APGAR) score of newborns at 10 minutes (min) were also measured. The reference ranges for thyroid function parameters in pregnant women were trimester-specific [21]. For TSH during pregnancy, we used trimester-specific ranges: 0.59–3.54 mIU/L (1^st^ trimester), 0.80–4.46 mIU/L (2^nd^ trimester), and 0.72–4.19 mIU/L (3^rd^ trimester) [22]. For FT3 during pregnancy, the trimester-specific reference ranges were as follows: 3.80–5.99 pmol/L (1^st^ trimester), 3.51–5.28 pmol/L (2^nd^ trimester), and 3.31–5.17 pmol/L (3^rd^ trimester) [23]. For FT4 during pregnancy, the following trimester-specific reference ranges were used: 11.8–18.4 pmol/L (1^st^ trimester), 11.6–17.4 pmol/L (2^nd^ trimester), and 9.7–15.1 pmol/L (3^rd^ trimester) [22].

A <3% of neonatal TSH values >5 mIU/L was used to determine iodine sufficiency in populations [3]. Low birth weight (LBW) was defined as a birth weight of <2500 g according to the definition of WHO [24]. All the biochemical analyses were performed using a Roche Cobas E601 according to the manufacturer's instructions. Blood samples of the same pregnant women were analysed in the same batch.

### 2.4. Statistical Analysis

Statistical analysis was conducted using SPSS ver. 16.0 (SPSS, Chicago, IL). The results were presented as mean ± standard deviation (SD) for quantitative variables. A paired *t*-test was used to analyse the continuous variables. In addition, a Chi-square test was used to analyse the categorical variables. One-way analysis of variance (ANOVA) was used to determine the difference in quantitative variables among different BMI categories. A *P* value <0.05 was considered as statistically significant.

## 3. Results

### 3.1. Participant Characteristics

A total of 3060 pregnant women consented to take part in the survey, and of these, 56.5% (*n* = 1729) were in the 2^nd^ trimester of pregnancy.  Table 1 shows the sociodemographic characteristics and biochemical results of participants. The overall mean age of participants was 28.2 ± 4.1 years, and the age range was from 18 to 45 years. All participants were of Han ethnicity.

### 3.2. Determination of Maternal TSH, FT3, and FT4

For the biochemical analysis, the overall mean value of TSH for participants was 1.88 ± 2.48 mIU/L (Table 1), which was within the normal reference range. There was no difference in mean TSH among the three trimesters of pregnancy (*P*=0.069). The overall mean value of FT3 for participants was 4.49 ± 1.51 pmol/L, which was within the normal reference range. The mean FT3 in 1^st^ trimester of pregnancy was significantly higher than those in 2^nd^ (*P*=0.001) and 3^rd^ trimesters of pregnancy (*P* < 0.001). The overall mean value of FT4 for participants was 15.91 ± 4.79 pmol/L, which was within the normal reference range. The mean FT4 in 1^st^ trimester of pregnancy was significantly higher than those in 2^nd^ (*P* < 0.001) and 3^rd^ trimesters of pregnancy (*P* < 0.001).

The overall prevalence of thyroid dysfunction in pregnant women was 12.9%. The prevalence of thyroid dysfunction in 1^st^ trimester of pregnancy (15.5%) was significantly higher than those in 2^nd^ (11.0%) and 3^rd^ trimesters of pregnancy (14.2%) (*P*=0.002).

### 3.3. Determination of Neonatal TSH, Birth Weight, and 10 min APGAR Score

The overall mean neonatal TSH was 4.86 ± 2.06 mIU/L. There were 29.3% of neonatal TSH values >5 mIU/L, which suggested an emergence of mild-to-moderate iodine deficiency in the region. The overall mean neonatal birth weight was 3358 ± 455 g. In addition, the overall prevalence of LBW was 3.2%. The overall mean 10-min APGAR score was 9.99 ± 0.21. According to the criteria of hypoxic-ischemic encephalopathy, the prevalence of 10-min APGAR score <5 was 0%.

There was no association between the prevalence of neonatal TSH values >5 mIU/L and LBW (*P*=0.740). Similarly, there was no difference in 10-min APGAR scores between the neonates with neonatal TSH values < 5 mIU/L and >5 mIU/L (*P*=0.541).

### 3.4. Maternal BMI

In terms of maternal BMI distribution, there were 48.1% (*n* = 1471) who were overweight followed by those in the obese (36.4%) and normal weight categories (15.5%) (Table 2). Participants who were obese had a significantly higher mean age than those who were in the normal weight (*P* < 0.001) and overweight categories (*P* < 0.001). Mean FT3 in the obese category was significantly higher than the normal weight category (*P* < 0.001) but not statistically different than the overweight category (*P*=0.123). In addition, mean FT4 in the overweight category was significantly higher than the obese category (*P*=0.022) but not statistically different than the normal weight category (*P*=0.931). Participants who were obese had a significantly higher neonatal birth weight than those who were in the normal weight (*P* < 0.001) and overweight categories (*P* < 0.001). There was no difference in mean TSH, mean neonatal TSH, prevalence of neonatal TSH values >5 mIU/L, and APGAR score among the three BMI categories (all *P* > 0.05).

### 3.5. Year of Study

The mean age of participants recruited in 2017 was significantly higher than those recruited in 2016 (*P* < 0.001) (Table 3). In addition, participants had a significantly higher mean FT4 value than those recruited in 2017 (*P*=0.003). There was no difference in weeks of gestation, average length of pregnancy (gestational age at birth), TSH, FT3, neonatal birth weight, neonatal TSH, and neonatal APGAR score at 10 min between the participants recruited in 2016 and 2017 (*P* > 0.05). The difference in neonatal TSH values >5 mIU/L between the two years was not statistically significant (i.e., 29.8% in 2016 and 28.9% in 2017) (*P*=0.592).

## 4. Discussion

This is one of the first large-scale observational studies that investigated the iodine and thyroid status of pregnant women and neonates in Jiangsu, China. Pregnant women and neonates are recommended for monitoring iodine status because they are more vulnerable to the adverse effects of iodine deficiency [25]. Pregnant women have a higher iodine requirement than nonpregnant adults (recommended iodine intake of 250 *μ*g/day vs. 150 *μ*g/day) so that they can achieve adequate iodine intake for the neurocognitive development of the fetus and maintain euthyroidism [3].

Neonatal TSH screening has been suggested to be used as an indicator to monitor for iodine deficiency in a population [3, 26]. In our study, the prevalence of elevated neonatal TSH (neonatal TSH values >5 mIU/L) was 29.3% in the region, suggesting the emergence of mild-to-moderate iodine deficiency. Similarly, a study conducted in Thailand reported that the prevalence of neonatal TSH values >5 mIU/L was 30.0%, 50.5%, and 58.5% in Bangkok, Nan, and Chiangmai, respectively [27]. Bangkok, Nan, and Chiangmai were iodine deficient areas with median UIC 64 *μ*g/L, mean UIC 49 *μ*g/g creatinine and 53 *μ*g/g creatinine, respectively [27]. Another multicentre study reported that the prevalence of neonatal TSH values >5 mIU/L was 32%, 52%, and 74% in the Philippines (median UIC 40 *μ*g/L), Malaysia (median UIC 33 *μ*g/L), and Kyrgyzstan (median UIC 30 *μ*g/L), respectively [28]. This is because when iodine intake is insufficient, the production of thyroid hormone is inadequate to maintain the development of central nervous function in neonates [28]. Subsequently, TSH value elevates in an attempt to stimulate the synthesis of thyroid hormone in neonates [28]. Therefore, our study confirmed the practical utility of neonatal TSH and its cutoff for assessing and monitoring iodine deficiency in China.

Our study reported no difference in mean TSH among the three trimesters of pregnancy, which is consistent with the findings reported by Liberman et al. [29]. The authors reported that an increase in serum TSH during gestation can be rectified by providing iodine supplements to pregnant women [29]. Only when iodine supply is limited, maternal iodine status will be affected [29]. Therefore, such findings are of interest but require further study. In addition, the genetics also play a role in normal thyroid function [30]. For example, in healthy individuals, thyroid function tests demonstrate substantial interindividual variability, suggesting that genetic factors are involved in the hypothalamus-pituitary-thyroid axis set point [31]. Mutation pathways identified in thyroid hormone synthesis have been shown to alter thyroid function [30]. However, further mechanisms of genetic influence such as iodothyronine deiodinase are yet to be fully discovered, particularly in thyroid disorders and autoimmune thyroid disease (AITD) [32].

### 4.1. A Re-Emergence of Iodine Deficiency?

Our findings were consistent with other study results that supported the presence of iodine deficiency among pregnant women in China [14, 33, 34]. These studies reported a decreasing trend of median UIC among pregnant women in China including Jiangsu [14, 33, 34]. In 2002, median UIC of pregnant women from urban and rural regions in Jiangsu was 247 *μ*g/L and 272 *μ*g/L, respectively [14, 33]. However, in 2014, the median UIC of pregnant women (*n* = 600) in Jiangsu was decreased to 155 *μ*g/L, and 23.7% of pregnant women had a median UIC <100 *μ*g/L [34], indicating that the iodine status of pregnant women was in the borderline iodine sufficiency. A median UIC of 150–249 *μ*g/L indicates iodine sufficiency in pregnant women [3]. Similarly, in other provinces of China including Zhejiang, pregnant women were reported to be mild-to-moderately iodine deficient [15–17].

Although the neonatal TSH can be used to assess iodine deficiency in populations [8, 35], there are still uncertainties regarding the cutoff values of neonatal TSH that are used to define the severity of iodine deficiency [36, 37]. In 1994, WHO/UNICEF/IGD included the criteria of using neonatal TSH values >5 mIU/L to define the severity of iodine deficiency [36]. According to the criteria recommended by WHO/UNICEF/IGD [36], the prevalence of neonatal TSH values > 5 mIU/L was divided into mild (3.0–19.9%), moderate (20.0–39.9%), and severe (≥40%). However, the criteria for defining the severity of iodine deficiency according to the prevalence of neonatal TSH values >5 mIU/L was not included in the newer versions published in 2001 and 2007 [3, 37]. Instead, WHO/UNICEF/IGD recommended that *a* <3% neonatal TSH values >5 mIU/L can be used to indicate iodine sufficiency when a sensitive TSH assay is used [3]. This is because there are several factors other than maternal iodine status which can affect neonatal TSH values, for example, the types of TSH assays, collection paper, timing of heel-prick blood sample collection, and the maternal or neonatal exposure to iodine-containing antiseptics at delivery [35]. Given the controversy with the recommendation by WHO/UNICEF/IGD [3], our study has provided some useful data to the current debate regarding the usefulness of neonatal TSH in assessing iodine deficiency in populations.

Iodised salt is the main source of dietary intake in China [13, 38]. Therefore, reducing the iodine intake from iodised salt could lead to a significant proportion of the population failing to achieve adequate iodine intake [13]. Decreased salt consumption has been reported in studies conducted in China in order to reduce the total salt intake for preventing noncommunicable diseases such as hypertension [39]. However, a decline in salt consumption will reduce the dietary iodine intake from table salt. Subsequently, this can potentially lead to a re-emergence of iodine deficiency in populations [40]. In fact, iodine deficiency during pregnancy has been reported in both developed and developing countries [41–47]. These countries include the UK [42], Belgium [43], Norway [45], Pakistan [47], Vietnam [41], Australia [46], and New Zealand [44]. Therefore, our study provided confirmation on this information and emphasized the need to monitor and surveillance iodine status of pregnant women in intervals.

The findings of our study have several strengths. We included 3060 mother-newborn pairs in our analysis, which was a large sample size. In addition, to the best of our knowledge, our study is the first study to examine the usefulness of the cutoff of <3% of neonatal TSH values >5 mIU/L to indicate iodine sufficiency in mother-newborn pairs in China after the new revised mandatory USI in 2012. However, our study was subject to a few limitations. First, we used a cross-sectional study design, and therefore a causal relationship could not be established. Second, the use of iodine supplement and dietary habits by pregnant women in our study were not recorded during their routine antenatal care. Third, we did not collect spot urine samples for the determination of UIC and used food frequency questionnaire (FFQ) in pregnant women. Therefore, our study was unable to assess iodine nutrition status in pregnant women. Although UIC is recommended by WHO/UNICEF/IGD to assess iodine status in pregnant women, [3] and iodine assessment in a different group of pregnant women with different TSH serum levels would reinforce our results, UIC only measures iodine intake within few days [48–50] and does not provide information on thyroid function [1, 49, 51]. In addition, during pregnancy, there is an increase of glomerular filtration rate which stimulates a higher daily urinary volume and might potentially lead to an overestimation of iodine deficiency in pregnant women [17]. Since our study only included healthy pregnant women, this might cause selection bias, and therefore our results should be interpreted with cautiousness.

In conclusion, our study suggested that there was an emerging iodine deficiency in the region according to the cutoff of <3% of neonatal TSH values >5 mIU/L proposed by WHO/UNICEF/IGD for the years evaluated. Although neonatal TSH can be used as biomarker of iodine deficiency in a population, it is important to point out that in our study, the limitation of drawing conclusions regarding pregnant women was just based on a neonatal parameter. A more comprehensive assessment of iodine status in pregnant women and neonates including the use of thyroglobulin (Tg) [52, 53] could be considered in order to complement the usefulness of neonatal TSH. The use of the cutoff <3% of neonatal TSH values >5 mIU/L as an indicator to suggest iodine sufficiency also requires further validation and standardisation, especially when and how to collect the neonatal blood samples after birth. Although IDD has been eliminated in Jiangsu since 2001, it is not impossible that iodine deficiency will emerge again in the region if corrective and preventive measures are not taken by the relevant health authorities. Also, at least in a smaller sample, we will try to verify and consider the prevalence of the most common thyroid diseases in the same region, while evaluating our results.

## Figures and Tables

**Table 1:** Sociodemographic characteristics and biochemical results of participants by trimesters.

	Trimesters			*P* value	Total (*n* = 3060)
	1st (*n* = 1183)	2nd (*n* = 1729)	3rd (*n* = 148)		
Age (years)	28.5 ± 4.0	28.2 ± 4.1	27.2 ± 4.0	0.001	28.3 ± 4.1
Weeks of gestation at recruitment	11.8 ± 2.1	17.2 ± 3.2	32.9 ± 3.3	<0.001	15.9 ± 5.4
TSH (mIU/L)	1.76 ± 3.35	1.94 ± 1.73	2.14 ± 1.41	0.069	1.88 ± 2.48
FT3 (pmol/L)	4.64 ± 1.08	4.43 ± 1.74	4.03 ± 1.43	<0.001	4.49 ± 1.51
FT4 (pmol/L)	16.90 ± 3.67	15.44 ± 5.42	13.51 ± 2.84	<0.001	15.91 ± 4.79

**Table 2:** Biochemical results of participants by maternal BMI.

	Maternal BMI			*P* value
	Normal (*n* = 475)	Overweight (*n* = 1471)	Obese (*n* = 1114)	
Age (years)	27.0 ± 3.3	28.2 ± 4.0	28.8 ± 4.3	<0.001
Weight (kg)	59.3 ± 4.2	68.2 ± 4.7	79.9 ± 7.7	<0.001
BMI	22.6 ± 1.1	26.0 ± 1.1	30.9 ± 3.9	<0.001
Average length of pregnancy (gestational age at birth)	38.9 ± 1.7	39.2 ± 1.5	39.1 ± 1.4	<0.001
TSH (mIU/L)	1.81 ± 1.48	1.89 ± 2.55	1.91 ± 2.71	0.771
FT3 (pmol/L)	4.25 ± 0.93	4.48 ± 1.41	4.60 ± 1.80	<0.001
FT4 (pmol/L)	16.17 ± 3.24	16.08 ± 4.14	15.58 ± 6.00	0.013
Neonatal TSH (mIU/L)	4.84 ± 1.81	4.85 ± 2.27	4.89 ± 1.84	0.840
Prevalence of neonatal TSH values >5 mIU/L (%)	27.6	29.0	30.5	0.466
Neonatal birth weight (g)	3150 ± 412	3342 ± 430	3469 ± 470	<0.001
Prevalence of LBW (%)	5.7	2.9	2.4	0.002
10-min APGAR score	9.98 ± 0.24	9.99 ± 0.17	9.99 ± 0.23	0.927

**Table 3:** Biochemical results of participants by year of study.

	2016 (*n* = 1657)	2017 (*n* = 1403)	*P* value
Age	27.9 ± 3.9	28.6 ± 4.2	<0.001
Weeks of gestation at recruitment	16.0 ± 5.3	15.7 ± 5.6	0.254
Average length of pregnancy (gestational age at birth)	39.1 ± 1.5	39.1 ± 1.5	0.130
TSH	1.82 ± 1.62	1.96 ± 3.21	0.115
FT3	4.53 ± 1.50	4.44 ± 1.53	0.091
FT4	16.14 ± 4.57	15.63 ± 5.03	0.003
Neonatal TSH (mIU/L)	4.89 ± 2.32	4.84 ± 1.69	0.512
Prevalence of neonatal TSH values >5 mIU/L (%)	29.8	28.9	0.592
Neonatal weight (g)	3373 ± 452	3342 ± 457	0.058
Prevalence of LBW (%)	2.8	3.6	0.253
Neonatal APGAR score	9.98 ± 0.24	9.99 ± 0.17	0.647

## Data Availability

The data used to support the findings of this study are available from the corresponding author upon request.
